# Vocal fold augmentation with autologous fat following transoral laryngeal surgery

**DOI:** 10.1007/s00405-025-09736-8

**Published:** 2025-10-28

**Authors:** Martin Sylvester Otte, Jens Peter Klussmann, Kevin Karl Hansen

**Affiliations:** 1 Department of Otorhinolaryngology, Head and Neck Surgery, University of Cologne, Medical Faculty, 50923 Cologne, Germany; 2 Center for Molecular Medicine Cologne (CMMC), University of Cologne, Faculty of Medicine and University Hospital Cologne, Cologne, Germany

**Keywords:** Transoral laser microsurgery, Injection laryngoplasty, Autologous fat grafting, Glottic insufficiency, Laryngeal scarring, Voice rehabilitation

## Abstract

**Background:**

Transoral laryngeal surgery (TOLS) may lead to glottic insufficiency and scarring which often causes pronounced hoarseness and sometimes swallowing impairment. Injection laryngoplasty with autologous fat is an established method to restore glottic competence and thus improve voice and swallowing function. We hereby aim to demonstrate how laryngeal fat injection can be applied following TOLS.

**Method:**

Autologous fat is harvested via liposuction and then centrifuged for three minutes at 3000 rpm (rpm) to separate fat cells from liquid fat and debris. Fat cells are then transferred to 1 ml Luer-Lock syringes and injected into the altered larynx via a 20 Gauge (0,9 mm) diameter injection needle under microscopic or endoscopic view. The primary objective hereby is approximation of remaining tissue on the glottic plain. The secondary objective is injection into areas of scarified tissue due to the beneficial effect of Adipose-Derived Mesenchymal Stem Cells and possible restoration of physiological laryngeal tissue properties.

**Summary:**

Injection laryngoplasty with autologous fat tissue may improve laryngeal function after partial laryngeal resection by laser surgery. Special attention needs to be paid to the specific individual postoperative anatomy of the patient’s larynx as this may differ profoundly from patient to patient.

**Supplementary Information:**

The online version contains supplementary material available at 10.1007/s00405-025-09736-8.

## Key points

1. Hoarseness after transoral laser surgery of the larynx is often caused by a residual gap during phonation.
2. Autologous fat tissue is highly suitable for medialization procedures of the larynx.
3. Thyroplasty type I is an established option for reducing glottic insufficiency, but may prove complex in post-TOLS patients
4. Fat may be harvested from peri-umbilically at a reasonably low risk.
5. After centrifugation, the lipid phase containing mature adipocytes is injected into the endolarynx.
6. A surgical microscope, exoscope or endoscope may be used for visualisation
7. If possible, injection should be performed laterally at the level of the glottic plain. It may be performed at the height of the vestibular folds, if residual tissue at the level of the glottic plain proves insufficient.
8. Scar tissue can make injection more difficult and lead to abrupt extrusion.
9. Injection into the paraglottic space is highly unlikely to cause breathing impairment for the patient.
10. Voice diagnostics and laryngo-stroboscopy should be performed both pre- and postoperatively in accordance with ELS guidelines.

## Background

Transoral laryngeal surgery (TOLS) is the standard treatment for small and medium-sized laryngeal carcinomas [1]. Despite the organ-preserving character, patients often complain of a deterioration in their voice, particularly after resection of glottic laryngeal carcinomas [2]. This is mainly due to a residual phonatory gap caused by tissue defects and scarring. Various surgical procedures are available for treatment of a phonatory gap, such as thyroplasty or laryngoplasty with rotation of the arytenoid [3], but these are complex and usually involve a transcervical approach. Injection laryngoplasty with autologous fat tissue however is a simple and often effective alternative.

## Relevant surgical anatomy

Exposure of the glottis is as in standard microlaryngoscopy. The area with the most relevant tissue defects needs to be exposed. The surgeon or an assistant can exert slight pressure on the larynx from the outside. The individual postoperative anatomy of each patient’s larynx may differ profoundly from patient to patient depending on the extent of previous resection. Subepithelial scaring may make fat application more difficult.

## Description of the technique

Subcutaneous fatty tissue is harvested from the lower abdomen using a 2 mm diameter liposuction cannula for fat harvesting (VoiceInject, Spiggle & Theiss). The collected fatty tissue is centrifuged at 3000 rpm for the duration of 3 minutes to separate fat cells from liquid fat and debris. Fat cells are transferred to 1 ml Luer-Lock syringes to be used for augmentation. When possible, augmentation is performed via injection into the paraglottic space via 20 G (0,9 mm) diameter injection needle (VoiceInject, Spiggle & Theiss). The injection site should always be adapted to the specific anatomy of each patient’s glottis, with the aim of reducing the glottic gap. Injection is therefore mainly performed laterally of the largest extent of soft tissue defects to medialize residual glottic tissue. Injection into areas of obvious scarring may also be performed in an attempt to improve tissue properties. Injection into a healthy contralateral vocal cord, when present, may also contribute to reducing the glottic gap.

## Indications

Injection laryngoplasty with autologous fat following TOLS is particularly suitable for patients with hoarseness due to a small to medium-sized glottal gap during phonation, which can be treated adequately with the material injected. However, the fat depot can also be applied in the area of the pocket folds or the arytenoid joints if the patient uses these structures for phonation after extensive surgery on the vocal folds. Reducing the residual glottic gap is also useful to prevent aspiration and improve coughing efficacy [4].

## Limitations

Due to varying extents of resection, injection laryngoplasty is not suitable for all patients who have undergone TOLS. Extensive tissue defects of the glottic plain cannot or not sufficiently be repaired by fat injection, resulting in persistent hoarseness. The injection of fat can be impaired by scar formation in the subepithelial tissue following laser surgery or hindered by an extensive tissue defect, resulting in simply not enough remaining tissue into which injection can be performed. Also, fat extrusion is an issue due to the diminished flexibility of scar tissue. Even with sufficient reduction of the glottic gap, an optimal voice usually cannot be directly achieved due to the loss of mucosal wave movement. Patients need to be counseled concerning these limitations.

## How to avoid complications

A challenge in injection laryngoplasty following TOLS is the rigidity of the scar tissue, which can lead to rupturing of the tissue. To avoid this, the fat should be applied slowly and under constant visual and tactile control. In our experience the medialization of the vocal cords does not induce dyspnea if sufficient space remains in the posterior part of the glottis. To avoid periumbilical infection after liposuction, a standard sterile work-technique is sufficient.

## Specific perioperative considerations/specific patient information

Preoperative diagnostics in accordance with the guidelines of the European Laryngological Society [5] are recommended. Laryngo-stroboscopy allows preoperative determination of optimal injection site and allows the expected benefit to be discussed with the patient. In the case of pronounced scarring or a severe residual gap during phonation, a thyroplasty type I via transcervical approach may be considered.

## Outcomes

The durability of fat augmentation remains a matter of debate in the literature, with some authors considering autologous fat as a permanent material and others as a temporary filler [6]. In our experience, long-term results vary considerably between patients. Notably, patients undergoing injection laryngoplasty following TOLC appear to benefit particularly well from the procedure, even in the long-term course. The beneficial effect may in part be related to adipose-derived mesenchymal stem cells contained in the graft [7]. The complication rate in our cohort has been very low; in particular, relevant airway compromise leading to dyspnea is not to be expected.

## Supplementary Information

**ESM 1.**
